# Canine Filarial Infections in a Human *Brugia malayi* Endemic Area of India

**DOI:** 10.1155/2014/630160

**Published:** 2014-05-20

**Authors:** Reghu Ravindran, Sincy Varghese, Suresh N. Nair, Vimalkumar M. Balan, Bindu Lakshmanan, Riyas M. Ashruf, Swaroop S. Kumar, Ajith Kumar K. Gopalan, Archana S. Nair, Aparna Malayil, Leena Chandrasekhar, Sanis Juliet, Devada Kopparambil, Rajendran Ramachandran, Regu Kunjupillai, Showkath Ali M. Kakada

**Affiliations:** ^1^Department of Veterinary Parasitology, College of Veterinary and Animal Sciences, Pookode, Wayanad, Kerala 673576, India; ^2^Department of Veterinary Pharmacology and Toxicology, College of Veterinary and Animal Sciences, Pookode, Wayanad, Kerala 673 576, India; ^3^Department of Animal Husbandry, Kerala 695 033, India; ^4^Department of Veterinary Anatomy, College of Veterinary and Animal Sciences, Pookode, Wayanad, Kerala 673 576, India; ^5^National Centre for Diseases Control, Kerala 673 003, India

## Abstract

A very high prevalence of microfilaremia of 42.68 per cent out of 164 canine blood samples examined was observed in Cherthala (of Alappuzha district of Kerala state), a known human *Brugia malayi* endemic area of south India. The species of canine microfilariae were identified as *Dirofilaria repens, Brugia malayi*, and *Acanthocheilonema reconditum*. *D. repens* was the most commonly detected species followed by *B. pahangi*. *D. immitis* was not detected in any of the samples examined. Based on molecular techniques, microfilariae with histochemical staining pattern of “local staining at anal pore and diffuse staining at central body” was identified as *D. repens* in addition to those showing acid phosphatase activity only at the anal pore. Even though *B. malayi* like acid phosphatase activity was observed in few dogs examined, they were identified as genetically closer to *B. pahangi*. Hence, the possibility of dogs acting as reservoirs of human *B. malayi* in this area was ruled out.

## 1. Introduction

Major filarial parasites of dogs are* Acanthocheilonema reconditum, Acanthocheilonema dracunculoides, Brugia malayi, Brugia pahangi, Brugia ceylonensis, Brugia patei, Cercopithifilaria grassii, Dirofilaria immitis*, and* Dirofilaria repens* [1–4]. Microfilariae of* Brugia* spp. are sheathed while those of others are not sheathed.

Canine filariosis was reported from various parts of India mainly from states like Kerala, Tamil Nadu, Karnataka, Orissa, West Bengal, Bihar, Uttar Pradesh, and Maharashtra. The species of microfilariae detected from these states include* D. immitis* from Kerala [5],* C. grassi* from Tamil Nadu [6],* D. immitis* from Himalayas  [7],* D. immitis* and* A. reconditum* from West Bengal [8],* D. immitis* and* D. repens* from Orissa [9],* D. repens* from Kerala [10],* D. repens* and* A. reconditum* from Karnataka [11],* A. reconditum, D. immitis, and D. repens *from Maharashtra and New Delhi, * and Microfilaria auquieri* and a novel species of* Acanthocheilonema* from Ladakh, India [12]. In general, it is believed that* D. immitis* is mostly prevalent in north eastern India [13] while* D. repens* is confined to southern parts of the country [14, 15].

Currently, there is paucity of information on the prevalence of filarial worms of dogs in the genus* Brugia* from India. The disease caused in humans by subperiodic* B. malayi*, in Malaysia and Indonesia, is considered zoonotic due to the existence of animal reservoir hosts like cats and dogs [16]. Brugian filariosis is endemic in Alappuzha district of Kerala State [17–19] and thus the presence of* B. malayi* in dogs is always suspected. Even though* B. malayi* like microfilariae [20, 21] were detected from cats and dogs from India, they were not confirmed as* B. malayi*. The present study focuses on the detection and differentiation of microfilariae of domestic canines inhabiting a* B. malayi* endemic area of southern India (Alappuzha district of Kerala state) based on histochemical staining and molecular techniques.

## 2. Materials and Methods

### 2.1. Ethics Statement

All study procedures and protocols were approved by the institutional animal ethics committee of College of Veterinary and Animal Sciences, Pookode, Kerala (which follows committee for the purpose of control and supervision of experiments on animals (CPCSEA) guidelines, Ministry of Environment and Forest, Government of India). The committee did not deem it necessary for this research group to obtain formal approval to conduct this study. National Centre for Diseases Control, directly under Directorate General of Health Services, Ministry of Health and Family Welfare, Government of India, is authorized to collect microfilaria positive human blood samples. Written informed consent was obtained from the human patients and owners of dogs before collection of samples.

### 2.2. Preliminary Screening and Collection of Samples

Samples were collected from dogs by collaborating veterinarians at “prophylactic antirabies vaccination and blood parasite detection camps” in the south Indian panchayats of Kerala state, namely, Kuthiathode (KUT), Kadakkarappally (KAD), and Pattanakkad (PKT). Brugian filariosis is endemic in this area [17–19]. Before sample collection data regarding the age, sex, and breed of these dogs were recorded.

Blood samples were collected from the saphenous vein. A drop of blood was examined by wet film technique at the camp site itself. Thick smears were prepared, fixed with methanol, stained using Giemsa, and examined for the presence of microfilariae with or without sheath. Whole nonheparinized (1 mL) and heparinized (1 mL) blood samples were also collected from the saphenous vein of all these dogs. A total of 164 blood samples of dogs (37 from Kadakkarappally, 57 from Kuthiathode, and 70 from Pattanakkad) were collected.

Blood sample of a known* B. malayi* infected human patient from Alappuzha, the endemic district of Kerala, was also collected to serve as positive control for histochemical staining and PCR techniques.

### 2.3. Histochemical Differentiation

The nonheparinized blood was allowed to clot and the serum fraction was centrifuged in an Eppendorf tube for 5 minutes at 1000 rpm. After discarding the supernatant, the sediment containing the microfilariae was resuspended in the remaining serum and a drop of this fluid was used for preparation of smears. They were air dried, fixed in absolute chilled acetone for 1 minute, further air dried, and stored in −20°C until used. These smears were used for histochemical staining within two weeks after preparation.

Histochemical staining was performed [22] to study the differences in the acid phosphatase enzyme activity in microfilariae for identification of the species  [22–26]. Briefly, 20 mL of solution I (Michaelis Veronal Acetate Buffer, pH 10.0) was mixed with 50 mL of distilled water and 4 mL of solution II (0.05 g Naphthol AS-TR phosphate, sodium salt (Sigma) in 5 mL of N, N-dimethyl formamide) was added to it in a beaker. In another beaker, 3.2 mL each of solutions III (1.0 g Pararosaniline hydrochloride, 5.0 mL concentrated hydrochloric acid, and 20.0 mL distilled water) and IV (4 per cent sodium nitrite) was mixed and then added to the mixture in the first beaker. The pH of the mixture was adjusted to 5.0 with 0.1 N sodium hydroxide solution. The final solution was prepared fresh every time before the staining procedure.

The air dried smears were incubated in the substrate for 1 hour at 37°C, rinsed in distilled water, counterstained in solution V (mixture of 77.1 mL of 0.2 M sodium phosphate, 122.9 mL 0.1 M nitric acid, and 2 g methyl green) for 5 minutes, and rinsed in distilled water. The slides were then dehydrated in 95 per cent and absolute ethyl alcohol, respectively, rinsed in xylene, and mounted using DPX. The smears were examined for the precipitated red azo dye indicating acid phosphatase activity.

### 2.4. Polymerase Chain Reaction

DNA isolation from the heparinised whole blood samples of dogs and human was based on phenol-chloroform isoamyl alcohol (PCI) method [27]. The leukocyte DNA from a three-day-old pup served as negative control. For the* Brugia* specific PCR, the DNA isolated from a known* B. malayi* infected human patient's blood served as positive control. Canine blood samples with heavy (one microfilaria in every low power field) monoinfection with* D. repens* and* A. reconditum* (confirmed by histochemical staining) served as positive controls for these species. The reactions were carried out in a thermal cycler (Eppendorf, Germany); the products were visualized (Alpha Innotech, USA) after electrophoresis on a 1.5 per cent agarose gel and documented.

#### 2.4.1. *D. repens* Specific PCR

PCR assay for the amplification of 246 bp direct tandem repeats of* D. repens* using specific primers [28] 5′-CCGGTAGACCATGGCATTAT-3′ (Forward) and 5′-CGGTCTTGGACGTTTGGTTA-3′ (Reverse) custom synthesized from IDT, USA, was standardized. The PCR amplification was performed in 25 *μ*L reaction volume containing 2.5 *μ*L 10x PCR buffer, 1 *μ*L (0.25 mM) dNTP, and 30 pmol of each primer, 1.5 U* Taq* polymerase, and 5 *μ*L of template DNA. Reaction conditions were as follows:  after the initial denaturation at 94°C for 5 minutes,  40 cycles each with 94°C for 30 seconds, 55°C for 30 seconds, and 72°C for 25 seconds were run.  A final extension at 72°C for 5 minutes was also given.  One product of* D. repens* specific PCR was directly sent to Chromos Biotech, Bangalore, for sequencing.

#### 2.4.2. *A. reconditum* Specific PCR

PCR assay for the amplification of 348 bp ITS2 fragment of* A. reconditum* using primers [29] 5′-CAGGTGATGGTTTGATGTGC-3′ (Forward) and 5′-CACTCGCACTGCTTCACTTC-3′ (Reverse) custom synthesized from IDT, USA, was standardized.**  **The PCR amplification was performed in 25 *μ*L reaction volume containing 2.5 *μ*L 10x PCR buffer, 1 *μ*L (0.25 mM) dNTP, and 0.3 mM of each primer, 2.5 U* Taq* polymerase, and 5 *μ*L of template DNA. The PCR cycling consisted of a denaturation step at 94°C for 3 minutes and 30 cycles each with denaturing at 94°C for 3 minutes, annealing at 63°C for 1 minute, extension at 72°C for 30 seconds, and final extension at 72°C for 7 minutes. One product of* A. reconditum* specific PCR was directly sent to Chromos Biotech, Bangalore, for sequencing.

#### 2.4.3. *Brugia* Specific PCR

PCR amplification of a 322 bp* Hha*1 fragment [30] using specific primers 5′-GCGCATAAATTCATCAGC-3′ (Forward) and 5′-GCGCAAAACTTAATTACAAAAGC-3′ (Reverse) constituted the genetic diagnostic criterion for* Brugia* infection in the study dogs. The PCR amplification was performed in 25 *μ*L reaction volume containing 2.5 *μ*L 10x PCR buffer, 1 *μ*L (0.25 mM) dNTP, and 10 pmol of each primer, 2.5 U* Taq* polymerase, and 5 *μ*L of template DNA. The PCR procedure consisted of an initial denaturation step at 94°C for 5 minutes and 35 cycles each of denaturation at 94°C for 1 minute, annealing at 56°C for 1 minute, extension at 72°C for 1 minute, and final extension at 72°C for 10 minutes. PCR products were cloned and sequenced.

#### 2.4.4. Amplification of 5.8S-ITS2-28S

In order to confirm the identity of microfilariae with new acid phosphatase staining pattern, PCR assay for the amplification of 5.8S-ITS2-28S fragment of rDNA gene ofsuspected samplesusing specific primers [3], 5′-AGTGCGAATTGCAGACGCATTGAG-3′ (Forward), 5′-AGCGGGTAATCACGACTGAGTTGA-3′ (Reverse), was standardized. The PCR amplification was performed in 25 *μ*L reaction volume containing 2.5 *μ*L 10x PCR buffer, 1 *μ*L (0.25 mM) dNTP, and 100 pmol of each primers, 1.5 U* Taq* polymerase, and 5 *μ*L of template DNA. Reaction conditions were as follows: after the initial denaturation at 94°C for 2 minutes, 32 cycles each with 94°C for 30 seconds, 60°C for 30 seconds, and 72°C for 30 seconds were run. A final extension at 72°C for 7 minutes was also given. PCR products were cloned and sequenced.

### 2.5. Cloning and Sequencing of PCR Products

PCR products were eluted from the gel using QIA quick gel extraction kit (QIAgen, Germany) based on manufacturer's protocol. The eluted product (8 *μ*L) was used for cloning into a pTZ57R/T vector (InsTAclone PCR cloning kit, Fermentas, USA) which was used to transform the JM107 strain of* E. coli* (Fermentas, USA). The transformed cells were plated on an LB agar with ampicillin (50 *μ*g/mL) as the selective antibiotic. After overnight incubation at 37°C, positive white colonies were selected and subcultured in LB broth overnight. The presence of the insert was confirmed by restriction digestion of the isolated plasmid from the subculture using* Eco*R1 and* Sal* I enzymes (GeneJET plasmid mini prep kit, Fermentas, USA). The stab culture of the colony containing the insert was sent for automated sequencing to Chromos Biotech, Bangalore. In order to verify mixed infection or recombination, 2 clones from each PCR products were sequenced.

### 2.6. Sequence Analysis

The sequence analysis was done using ClustalV method of MegAlign programme (DNASTAR, USA).

## 3. Results

### 3.1. Preliminary Screening

A total of 164 blood samples of dogs were collected from Kadakkarappally (37), Kuthiathode (57), and Pattanakkad (70). Out of 164 dogs, 114 were from nondescript animals while 50 were from exotic breeds (Spitz, German shepherd, Dachshund, and Doberman).

Wet film examination revealed microfilariae in 11 (29.72 per cent) samples from Kadakkarappally, 23 (40.35 per cent) from Kuthiathode and 17 (24.29 per cent) from Pattanakkad (Table 1). The overall prevalence of microfilaremia based on wet film examination was 31.17 per cent.

Giemsa staining technique revealed microfilariae in 16 (43.24 per cent) samples from Kadakkarappally, 29 (50.87 per cent) from Kuthiathode, and 25 (35.71 per cent) from Pattanakkad. Overall, the prevalence of microfilaremia based on Giemsa staining was 42.68 per cent. Four dogs revealed microfilariae with sheath.

Fifty two per cent of exotic breeds and 38 per cent of nondescript breeds of dogs harboured microfilariae. Prevalence of microfilaraemia was more common in dogs above two years of age. Also, male dogs exhibited higher prevalence (Table 2).

### 3.2. Histochemical Differentiation

**Figure 1:**
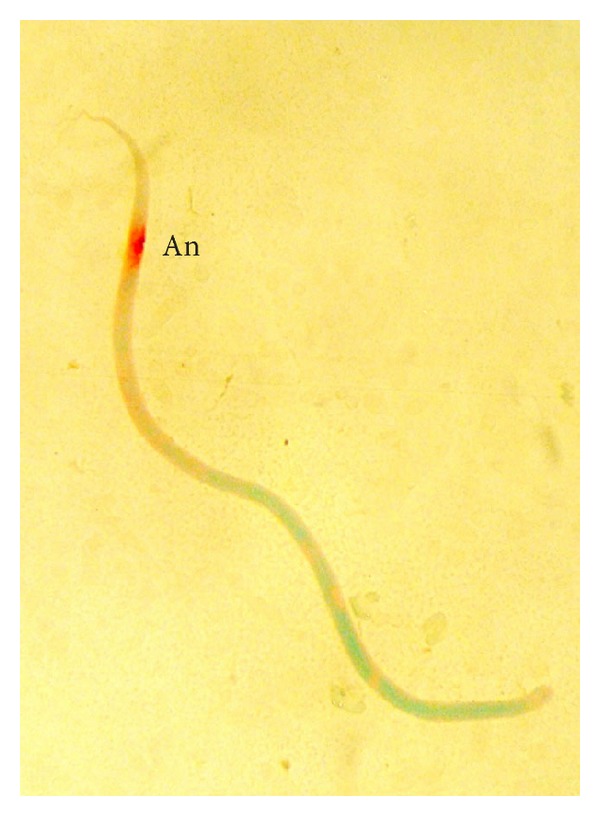
*D. repens* microfilaria showing acid phosphatase activity at the anal pore (An).

The* D. repens* microfilariae showed single locus of intense acid phosphatase staining in the region of the anal pore (Figure 1). This type of reaction was observed in 27.4 per cent of dogs.

**Figure 2:**
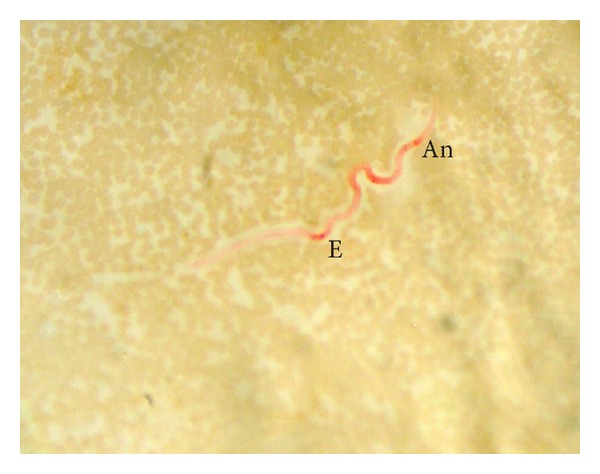
*A. reconditum* microfilaria showing acid phosphatase activity throughout the entire body, especially between the excretory (E) and anal pores (An).

The entire body, especially between the excretory and anal pores, was stained bright red in microfilariae of* A. reconditum* (Figure 2). Only two out of 164 smears examined revealed this type of reaction.

**Figure 3:**
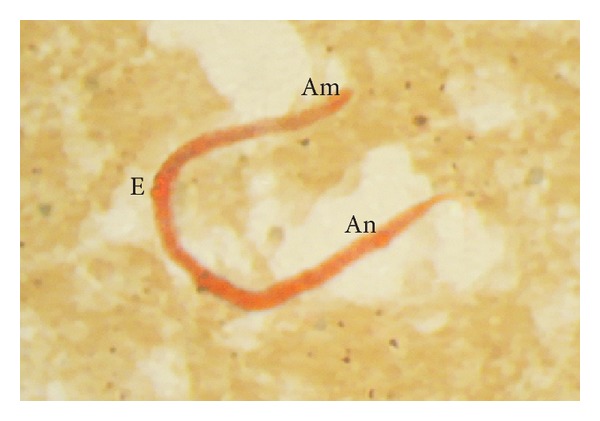
*B. pahangi* microfilaria showing heavy and diffuse acid phosphatase activity along the entire body length. The excretory (E) and anal pores (An) are recognizable.

For* B. pahangi* microfilariae, the heavy and diffuse acid phosphatase activity was observed along the entire body length, although the excretory and anal pores were still recognizable (Figure 3). A total of 18 smears (11 per cent) showed this type of reaction.

**Figure 4:**
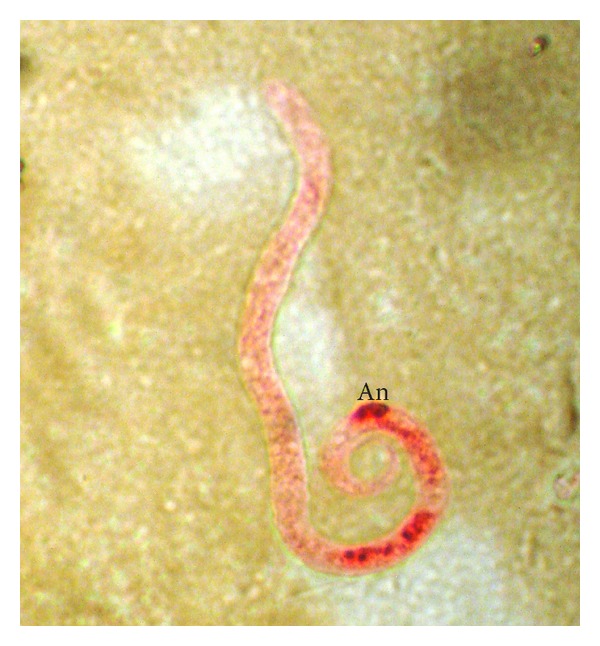
Microfilaria showing acid phosphatase activity with red spot at anal pore (An) region and diffuse red staining at the central body.

The acid phosphatase staining pattern, with local staining at the anal pore and diffuse staining in the central body (Figure 4), was observed in 58 cases (35.4 per cent). Most of the samples contained a mixture of* D. repens* and this type of microfilariae.

**Figure 5:**
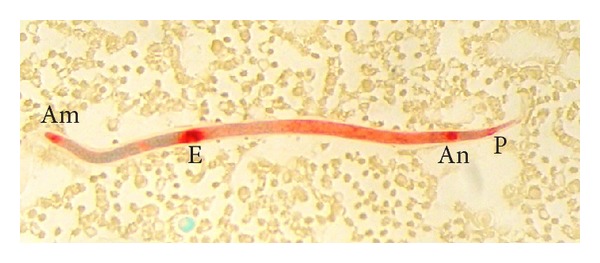
Human* B. malayi* microfilaria showing acid phosphatase activity in the areas of amphid (Am), excretory pore (E), anal pore (An), and phasmid (P).

**Figure 6:**
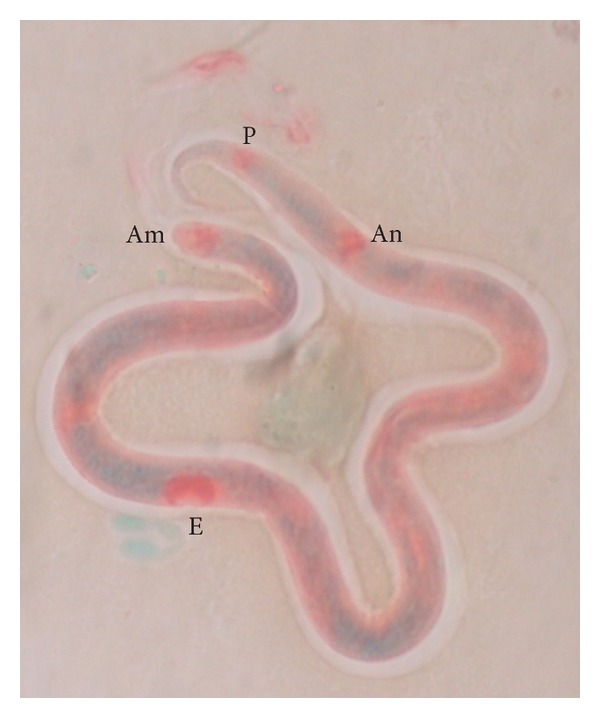
Canine* B. malayi* like microfilaria showing acid phosphatase activity in the areas of amphid (Am), excretory pore (E), anal pore (An), and phasmid (P).

Blood smears prepared from human patients with confirmed* B. malayi* infection contained microfilariae with loci of acid phosphatase activity in the areas of the amphids, the excretory pore, the anal pore, and the phasmids (Figure 5). Out of 164 dogs tested, 6 harboured microfilariae with histochemical reaction similar to* B. malayi* (Figure 6).

### 3.3. Polymerase Chain Reaction

#### 3.3.1. *D. repens*

PCR detected the 246 bp* D. repens*-specific product in 64 out of 164 samples. PCR product from a sample that contained microfilariae showing a single locus of staining in the region of the anal pore was sequenced for confirmation (accession number JN830762).

#### 3.3.2. *A. reconditum*

PCR with primers specific for* A. reconditum* amplified the expected 348 bp product from two samples containing microfilariae with histochemical reactions typical of* A. reconditum*. The partial sequence of the PCR product was submitted to GenBank (accession number JQ039745).

#### 3.3.3. *Brugia *Species

PCR with primers specific for* Brugia* sp. amplified the expected 322 bp product from 23 (14 per cent) samples.

**Figure 7:**
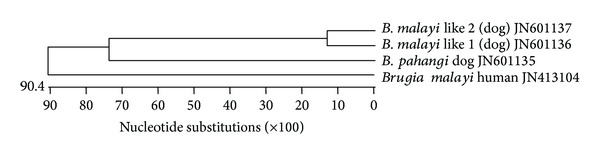
Phylogenetic tree constructed based on* Hha*1 sequence of different* Brugia* species.

**Figure 8:**
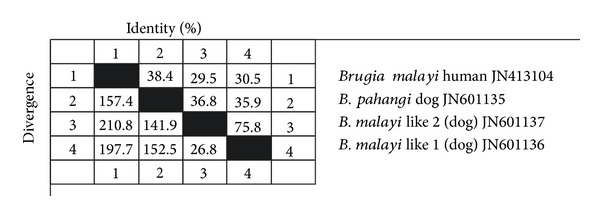
Distance matrix based on* Hha*1 sequence of different* Brugia* species.

One* Hha*1 PCR product each from samples showing only* B. pahangi* (PKT 94) (accession number JN601135), human* B. malayi* (accession number JN413104), and* B. malayi* like parasite of dog (two clones from KAD 19) (accession numbers JN601136 and JN601137) were cloned and sequenced. The phylogenetic tree (Figure 7) and distance matrix (Figure 8) plotted based on ClustalV method of MegAlign programme (DNASTAR, USA) demonstrated that the microfilariae with histochemical reaction similar to* B. malayi* were genetically closer to* B. pahangi* of dogs.

#### 3.3.4. Amplification of 5.8S-ITS2-28S

For confirmation of the species of microfilariae with the acid phosphatase staining reaction pattern “local staining at the anal pore and diffuse staining in the central body,” sequence analysis of PCR product of 5.8S-ITS2-28S fragment of rDNA gene was resorted to. The 584 bp fragment was sent for sequencing. When submitted to NCBI-BLAST, the 492/498 bp stretches of the 584 bp product (accession numbers JQ039743 and JQ039744) shared 97 per cent identity with the previously reported* D. repens* sequence AY 693808.

## 4. Discussion

Filarial infections in dogs can be diagnosed through morphological observations of the circulating microfilariae, detection of circulating antigens, histochemical staining of circulating microfilariae, or through molecular approaches. Proper identification of circulating microfilariae based on morphology requires the involvement of a well-trained parasitologist. Filarial infection with multiple species and morphological alterations of microfilariae due to incorrect preventive treatment of dogs are not easily differentiated morphologically even by trained persons [31].

Histochemical staining to detect acid phosphatase activity can overcome most of these problems; however, this technique requires fresh samples to yield optimal results. Besides being time consuming and labour intensive, both staining methods require expertise to identify and confirm the species [32]. Molecular methods based on species specific PCRs are simple and easy to perform. Use of properly designed primers and control template DNAs can make PCR assays species specific. PCR assays are very sensitive due to the exponential amplification of target DNA. Additionally, it may be possible with a PCR to detect circulating DNA liberated from host destroyed microfilariae or from adult worms [30].

Microscopical examination of Giemsa stained blood smears were used previously for detection of the presence of microfilaremia in dogs of Thrissur, Kerala. The prevalence varied from 7 to 26.5 per cent [10, 12, 33]. In the present study, using the same technique, the overall prevalence of microfilaremia in dogs was 42.68 per cent and the highest prevalence (50.87 per cent) was observed in Kuthiathode. Only two samples revealed sheathed microfilariae. Other techniques (histochemical staining and PCR) employed in the study detected more number of dogs positive for microfilariae especially* Brugia* species.

Even though the most pathogenic canine filaria,* D. immitis*, was reported previously from the Kerala state [5], it was not identified in any of the dogs tested in the present study. Unambiguous identification of* D. repens* and* A. reconditum* in dogs from the study population by histochemical staining and sequencing of PCR products was possible in this study.

Recently,* A. reconditum* (9.3 per cent) was identified as the most common species in North India [12] followed by* D. repens* (6.7 per cent) and* D. immitis* (1.5 per cent). In the present study, the microfilariae with a new histochemical staining pattern (local staining at the anal pore and diffuse staining at the central body) were more common. Based on 97 per cent identity of 5.8S-ITS2-28S region of rDNA gene [3] of this form of microfilariae with that of* D. repens*, the species was confirmed as* D. repens*. Therefore,* D. repens* is identified as the most common species of filarial worm in the study area. However, 97 per cent identity may also indicate an interspecific difference and therefore the possibility of a new species could not be ruled out.

Another important finding of the study was the presence of* Brugia* species in the dogs of the area. The most prevalent* Brugia* species identified in the study area was* B. pahangi*. Out of 164 dogs examined, 18 (11 per cent) harboured microfilariae with acid phosphatase staining pattern specific to that parasite. Histochemical staining pattern similar to* B. malayi* was observed in 6 out of 164 dogs examined. PCR specific for* Brugia* sp. (*Hha*1) revealed the diagnostic 322 bp product in 23 samples that included* B. malayi* like microfilariae and* B. pahangi* as indicated by histochemical staining.* Hha*1 PCR products amplified in samples of dogs showing only either of these parasites based on histochemical staining were selected for sequencing. Similarly, the* Hha*1 fragment of the* B. malayi* (human) was also sequenced. Phylogenetic analysis of the sequences revealed that the suspected* B. malayi* like parasite was genetically closer to* B. pahangi* (dogs) than to* B. malayi* (human). Therefore, the results of the present study did not indicate potential of dogs acting as reservoirs of* B. malayi*.

However, a recent study conducted at Thrissur, Kerala, (100 km away from the study area) revealed that out of 100 dogs with symptoms of filariosis (fever, anorexia, conjunctivitis, oedema of limb, and scrotum) circulating microfilariae were detected in 80 cases, of which all the 16 cases with sheathed microfilariae were identified as* B. malayi* based on histochemical staining and PCR [20]. The primers specific for amplification of 294 bp trans-spliced exon 1 (SLX) region (5S r RNA) of* Brugia* species [16] followed by sequence analysis (90 bases with query coverage of 32 per cent) was used for confirming the identification of* B. malayi* in dogs. The authors concluded that the high prevalence of* B. malayi* in Thrissur emphasized the possible role of dogs in the transmission of human filariosis. The results of the present study are contrary to the above report.

Lymphatic filariosis is one of the major diseases of mankind in tropical and subtropical countries and the global burden of this type of disease is 119.1 million cases [34] of which 12.9 million are affected by Brugian filariosis. In India,* B. malayi* was reported from a few foci only, the largest single tract being Travancore-Cochin state (present study area) [17].* B. malayi* is known to occur in two forms, periodic and subperiodic. The disease caused by subperiodic* B. malayi*, in Malaysia and Indonesia, is considered zoonotic due to the existence of animal reservoir hosts like cats and dogs [16, 35]. Previously, two out of 57 cats examined in Orissa state, India, were found infected with* B. malayi* like microfilariae [21].

Other epidemiological factors also should be considered before confirming the absence of reservoir status in dogs for* B. malayi* in Cherthala. The zoonotic subperiodic* B. malayi *is transmitted in nature by* Mansonia bonnae* while* M. dives, M. annulata*, and* M. uniformis* are efficient laboratory vectors. The Indian situation is different. Microfilariae of* B. malayi* in India are purely nocturnal in their periodicity [21]. In Cherthala “Taluk” (an administrative subdivision of a district) of Alappuzha district of Kerala, Brugian filariosis is transmitted by* M. annulifera, M. uniformis*, and* M. Indiana* [17–19]. The efficient vector (*M. bonnae*) for the transmission of zoonotic Brugian filariosis is not reported from this area. Also, a 90.7 per cent decline in the trend of disease rate for human filarial infections in Cherthala Taluk was also reported [36]. If animal reservoirs like dogs were there in existence, there would have been further increase in the number of outbreaks of the disease because of the availability of reservoirs (including stray dog population) and absence of microfilaricidal therapy in them. Also, when* B. malayi* could not be detected from Cherthala, the endemic hotspot, it is difficult to detect the parasite from a place which is 100 km away from the endemic area. So, the possibility for dogs acting as reservoirs of* B. malayi* in these areas is very remote. In addition, the clinical symptoms reported in dogs of Thrissur, Kerala [20], were similar to that of dogs infected with* B. pahangi* [37] which include varying levels of microfilaraemia, episodic lymphadenopathy, lymphangitis, and limb oedema. For these reasons, the detection of* B. malayi* in dogs of Thrissur might be a false detection.

The microfilariae of three species, namely,* B. tupiae* (reported previously from Malaysia, Thailand, and Vietnam),* B. ceylonensis*, and* B. pahangi*, are liable to be confused with those of* B. malayi* [38].* B. ceylonensis* was first described in lymphatics of dogs in 1962 from Sri Lanka [39]. This parasite, which is transmitted by* Aedes aegypti*, was reported from Kerala too as early in 1974, but there is no recent documentation of this parasite from the state. By contrast,* B. ceylonensis* was recently reported from the conjunctiva of a human patient in Sri Lanka [40]. Also, a Sri Lankan survey of 65 dogs revealed 7 per cent prevalence of single infection with* B. ceylonensis* [41]. The present study could not differentiate the* B. malayi* like microfilariae of dogs from* B. ceylonensis* due to the absence of reports on the typical histochemical staining reaction of* B. ceylonensis* microfilariae or gene accessions. The* B. malayi* like microfilariae observed in dogs of the study area could be a new* Brugia* species,* B. ceylonensis*, or a genetic variant of* B. pahangi*.

## 5. Conclusion

Microfilariae of* D. repens*,* A. reconditum*, and* B. pahangi* occur in dogs of Cherthala Taluk, Alappuzha district, Kerala, the human* B. malayi* endemic area of south India. Sequence analysis of 5.8S-ITS2-28S fragment of rDNA gene of microfilariae with the acid phosphatase staining pattern of local staining at the anal pore and diffuse staining in the central body revealed 97 per cent homology with* D. repens*. The* B. malayi* like microfilariae observed in dogs of the study area could be a new* Brugia* species,* B. ceylonensis*, or a genetic variant of* B. pahangi*.

## Figures and Tables

**Table 1:** Prevalence of canine microfilariae in a human *B. malayi* endemic area of Kerala, India, based on wet film examination, Giemsa staining, histochemical staining, and polymerase chain reaction.

Place	Samples examined	W. f	G	Histochemical reaction/acid phosphatase reaction					Polymerase chain reaction		
				*D. repens *	*A. reconditum *	*B. pahangi *	New reaction	*B. malayi * like	*D. repens *	*A. reconditum *	*Brugia* sp. (*Hha*1)
Kadakkarappally	37	11	16	11	0	3	11	3	10	0	6
Kuthiathode	57	23	29	18	2	3	28	0	28	2	7
Pattanakkad	70	17	25	16	0	12	19	3	26	0	10

Total	164	51	70	45	2	18	58	6	64	2	23

W. f: wet film examination; G: Giemsa staining.

**Table 2:** Microfilaraemia in different breeds, age groups, and sexes of dogs in a *B. malayi* endemic area of Kerala, India, based on Giemsa staining technique.

Blood smears	Area	Breed					Age group		Sex		Total
		ND	Exotic								
			Spitz	G.S.D	Dach	Dob	<2 years	>2 years	Male	Female	
Examined	Kadakkarappally	29	6	2	0	0	14	23	33	4	37
	Kuthiathode	36	10	5	4	2	13	44	39	18	57
	Pattanakkad	49	17	2	1	1	31	39	56	14	70
	Total	**114**	**33**	**9**	**5**	**3**	**58**	**106**	**128**	**36**	**164**
Positive	Kadakkarappally	11	3	2	0	0	4	12	16	0	16 (43.24%)
	Kuthiathode	16	6	2	3	2	4	25	24	5	29 (50.87%)
	Pattanakkad	17	6	1	0	1	3	22	20	5	25 (35.71%)
	Total	**44 (38.6%)**	**15 (45.46%)**	**5 (55.56%)**	**3 (60%)**	**3 (100%)**	**11 (18.97%)**	**59 (55.66%)**	**60 (46.88%)**	**10 (27.78%)**	**70 (42.68%)**

ND: Nondescript, G.S.D: German shepherd, Dach: Dachshund, Dob: Doberman.

## Abbreviations

DNA: Deoxyribonucleic acid
PCR: Polymerase chain reaction
rDNA: Ribosomal DNA
DPX: Distyrene, plasticiser (dibutyl phthalate), and xylene.
